# WTAP participates in neuronal damage by protein translation of NLRP3 in an m6A-YTHDF1-dependent manner after traumatic brain injury

**DOI:** 10.1097/JS9.0000000000001794

**Published:** 2024-06-14

**Authors:** Yuhua Chen, Tianlin Long, Junhui Chen, Hong Wei, Jiao Meng, Meili Kang, Juning Wang, Xin Zhang, Quanhua Xu, Chi Zhang, Kun Xiong

**Affiliations:** a Department of Neurosurgery, Bijie Traditional Chinese Medical Hospital, Bijie, Guizhou; b Department of Central Laboratory, Xi’an Peihua University, Xi’an, Shaanxi; c Department of Anatomy and Neurobiology, School of Basic Medical Science, Central South University, Changsha, Hunan; d Department of Neurosurgery, Wuxi Clinical College of Anhui Medical University, 904 Hospital of Joint Logistic Support Force of PLA, Wuxi, Jiangsu Province; e Department of Rehabilitation Teaching and Research, Xi’an Siyuan University, Xi’an; f Department of Neurosurgery, Xiangya Hospital, Central South University, Changsha; g Key Laboratory of Emergency and Trauma, Ministry of Education, College of Emergency and Trauma, Hainan Medical University, Haikou, Hainan; h Hunan Key Laboratory of Ophthalmology, Changsha, Hunan, People’s Republic of China

**Keywords:** N6-methyladenosine, neuronal damage, NLRP3, traumatic brain injury, WTAP, YTHDF1

## Abstract

**Background:**

Traumatic brain injury (TBI) is a common complication of acute and severe neurosurgery. Remodeling of N6-methyladenosine (m6A) stabilization may be an attractive treatment option for neurological dysfunction after TBI. In the present study, the authors explored the epigenetic methylation of RNA-mediated NLRP3 inflammasome activation after TBI.

**Methods:**

Neurological dysfunction, histopathology, and associated molecules were examined in conditional knockout (CKO) WTAP^[flox/flox, Camk2a-cre]^, WTAP^flox/flox^, and pAAV-U6-shRNA-YTHDF1-transfected mice. Primary neurons were used in vitro to further explore the molecular mechanisms of action of WTAP/YTHDF1 following neural damage.

**Results:**

The authors found that WTAP and m6A levels were upregulated at an early stage after TBI, and conditional deletion of WTAP in neurons did not affect neurological function but promoted functional recovery after TBI. Conditional deletion of WTAP in neurons suppressed neuroinflammation at the TBI early phase: WTAP could directly act on NLRP3 mRNA, regulate NLRP3 mRNA m6A level, and promote NLRP3 expression after neuronal injury. Further investigation found that YTH domain of YTHDF1 could directly bind to NLRP3 mRNA and regulate NLRP3 protein expression. YTHDF1 mutation or silencing improved neuronal injury, inhibited Caspase-1 activation, and decreased IL-1β levels. This effect was mediated via suppression of NLRP3 protein translation, which also reversed the stimulative effect of WTAP overexpression on NLRP3 expression and inflammation.

**Conclusions:**

Our results indicate that WTAP participates in neuronal damage by protein translation of NLRP3 in an m6A-YTHDF1-dependent manner after TBI and that WTAP/m6A/YTHDF1 downregulation therapeutics is a viable and promising approach for preserving neuronal function after TBI, which can provide support for targeted drug development.

## Graphical abstract

**Figure:**
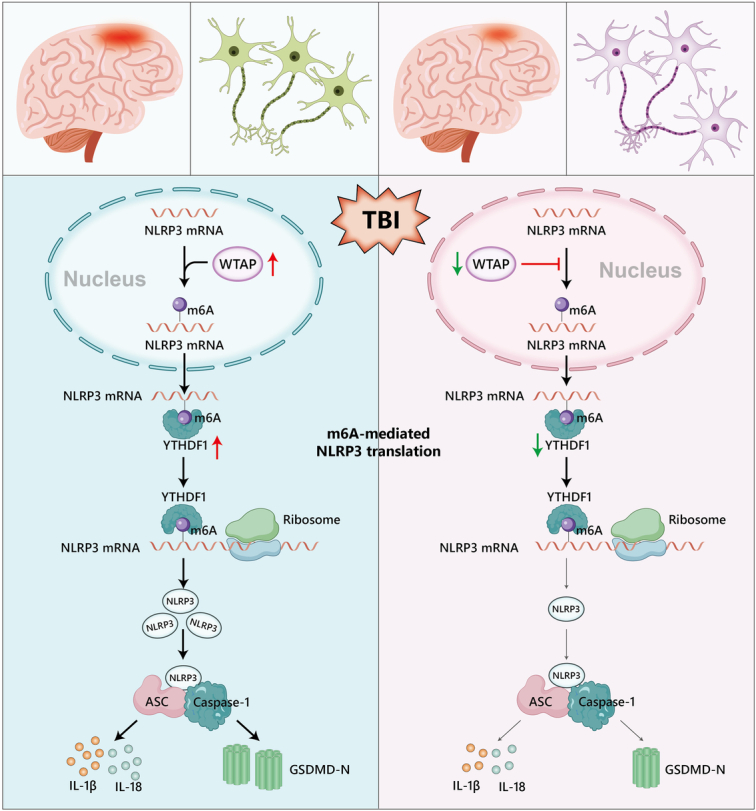

## Introduction

Highlights

- WTAP and m6A levels were upregulated at early stage after traumatic brain injury, and neuronal WTAP conditional knockout promoted functional recovery.
- WTAP regulates NLRP3 m6A-mediated neuroinflammation and neuronal damage.
- YTHDF1 downregulation and mutation reduced NLRP3 protein translation in neuronal injury.
- WTAP/m6A participates neuronal damage by NLRP3 protein translation in m6A-YTHDF1-dependent manner.

Traumatic brain injury (TBI) is a common neurosurgical emergency with high incidence, disability, and mortality rates^1^. Despite living in a relatively peaceful era, war and its associated risks persists in some parts of the world, remaining a significant cause of TBI^2^. Studies from the Afghanistan and Iraq wars indicate that TBI affects roughly 20% of the American soldiers^2^. And traffic, falls, falling objects and blows are all important factors that cause TBI^2^. The medical cost of mild TBI patients is 2–3 times that of non-TBI patients^1,3^, which brings heavy economic burden to the patients and their families as well as to the society. The main reason for this is that its pathogenesis remains unclear. In addition to brain injury caused by primary trauma, secondary brain injury is also the main cause of neurological dysfunction in the pathogenesis of TBI^4,5^. Currently, the mechanisms of secondary brain injury in TBI mainly include neuronal apoptosis, inflammation, mitochondrial dysfunction, and oxidative stress^5,6^. Although the management and treatment of patients with TBI have made great progress in recent years, effective treatment strategies are still lacking^1,7^. Therefore, further elucidation of the post-TBI pathological mechanisms and exploration of potential therapeutic targets could provide new possibilities for clinical treatment and prognostic judgment.

N6-methyladenosine (m6A), one of the most pervasive modifications in eukaryotic RNA^8,9^, is finely mediated by its ‘writer’ (METTL3, METTL14, WTAP, VIRMA, and ZC3H13), ‘reader’ (YTHDF1/2/3, YTHDF1/2/3, FMRP, and PRRC2A), and ‘eraser’ (ALKBH5 and FTO)^10–14^. m6A is more abundant in the nervous system than in other organs and its overall abundance increases from the embryonic brain to the adult brain, indicating that it is critical for the formation and execution of neural functions^11^. Aberrant m6A has been linked to several diseases^15–18^, such as depression^19^, Alzheimer’s disease (AD)^20^, Parkinson’s disease (PD)^21^, stroke^22^, and TBI^23^. Due to the complexity of the nervous system and network regulation, the role and mechanism of m6A in nervous system diseases remain to be elucidated. Several studies have investigated the transcriptional levels of m6A-related RNA methylation after TBI. Abnormal m6A modifications have been found in the hippocampus of mice and rats with TBI^24,25^, while the expression of METTL3 is downregulated in the hippocampus of mice following TBI^24^. Yu *et al*.^23^ explored the m6A regulation of cortical mRNA in rats and found that FTO intervention may be related to the improvement of neurological function post-TBI. Thus, the remodeling of m6A level stabilization may be an attractive potential treatment option for neurological dysfunction after TBI.

There are many forms of cell death following neuronal injury, and its regulation can contribute to the improvement of neural function^26,27^. The NLRP3 inflammasome, an important regulatory switch for pyroptosis, affects neuronal injury and neuroinflammation after TBI and is regarded as a promising therapeutic target for TBI^28,29^. Our previous studies aimed to reveal the function of the NLRP3 inflammasome in TBI pathological processes and explore its underlying endogenous regulatory mechanism^29–32^. METTL3-, METTL14-mediated RNA methylation, and m6A reader protein YTHDF1 has been reported to regulate NLRP3 expression in atherosclerosis^33^, arsenic-induced hepatic insulin resistance^34^, and sepsis^35,36^, respectively. However, m6A data for the NLRP3 inflammasome in the cerebral cortex following TBI and in patients with clinical TBI have not been reported. In this study, we explored the epigenetic methylation of RNA-mediated NLRP3 in the cerebral cortex of mice and its functions in nerve injury after TBI, which may reveal the underlying function and regulatory mechanism of m6A in controlling cortical nerve damage, pathological processes, clinical diagnosis, and further intervention in TBI.

## Materials

Ethical approval for this study was approved by the Animal Care and Use Committee, in accordance with the ARRIVE guidelines (Supplemental Digital Content 1, http://links.lww.com/JS9/C760)^37^. The floxed WTAP allele (WTAP^f/f^) C57BL/6J mice (purchased from Cyagen Biosciences Inc. (Guangzhou, China), then bred with neuronal conditional knockout (CKO) WTAP^[flox/flox, Camk2a-cre]^ mice using Camk2a-cre transgenic mice) and wild type (WT) mice (8 weeks, 20±2 g) were exposed to a 12 h/12 h light-dark cycle, along with free activity and foraging (including food and water).

The animals were anesthetized with 2–3% isoflurane inhalation anesthesia (RWD Life Science Co.; 1–1.5% maintain) and maintained to a normal temperature of mice using a thermostatic heating pad at 37° during and after surgery. The mice were held in a stereotaxic frame and the scalp was exposed and cleaned with povidone-iodine. TBI was induced using a controlled cortical injury (CCI) device (RWD Life Science Co.) as described in our previous study^32,38^.

For YTHDF1 knockdown (KD) mice, YTHDF1 pAAV-U6-shRNA (targeting sequence: GCTGAAGATTATCGCTTCCTA; Beijing Syngenbio Co., Ltd.) and negative control (NC) pAAV-U6-shRNA were prepared according to the manufacturer’s instructions. The right lateral ventricles (depth: 3.0 mm) were injected with 2 μl 10^12^ VG/ml virus (targeting YTHDF1 or NC) at 1 μl/min; the needle remained in place for 2 min and was slowly recovered once the injection was complete. Follow-up experiments were conducted 2 weeks later.

Other detailed experimental procedures, procedures, and statistical analysis were shown in the Supplementary Materials, Table S1 (Supplemental Digital Content 2, http://links.lww.com/JS9/C761) and Supplementary Materials Figure S1-S5 (Supplemental Digital Content 2, http://links.lww.com/JS9/C761).

## Results

### TBI enhances m6A level and WTAP expression

**Figure 1:**
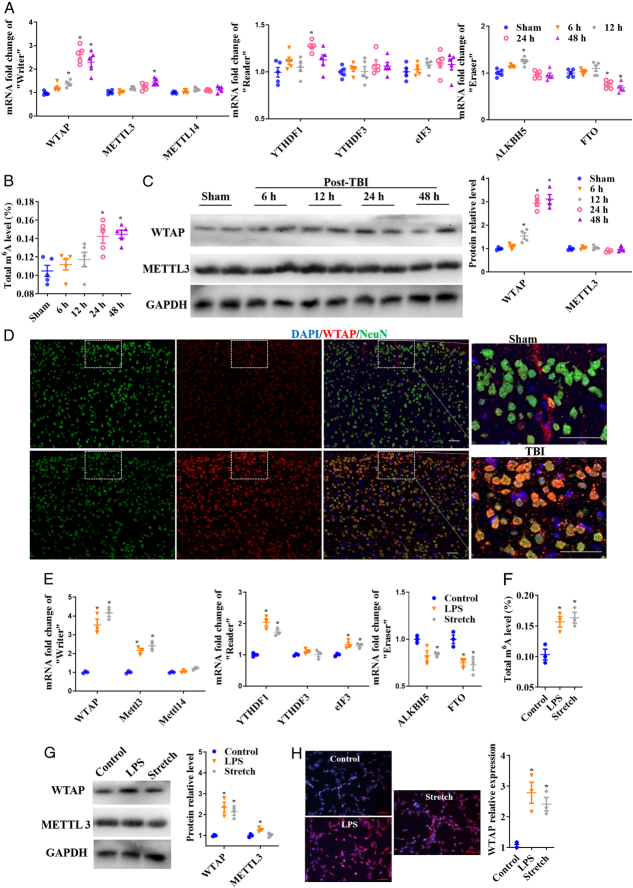
m6A level and WTAP expression were increased after TBI and neuronal injury in vitro. (A) Expression of m6A ‘Writers’, ‘Erasers’, and ‘Readers’ quantified by qRT-PCR in mouse cerebral cortex after TBI. (B) Total m6A levels quantified using EpiQuik m6A RNA Methylation Quantification Kit. (C) WB analysis of WTAP and METTL3 expression in mouse cerebral cortex after TBI. The results were statistically analyzed. (D) IF analysis of WTAP expression in mouse cerebral cortex at 48 h after TBI, scale bars =50 μm. Data are presented as the mean±SEM (*n*=5). *P<0.05, *versus* sham group. (E) Expression of m6A ‘Writers’, ‘Erasers’, and ‘Readers’ quantified by qRT-PCR in LPS+ATP-induced and stretch-induced primal cortex neuronal injury. (F) The total m6A level measured in primal cortex neurons. G WB analysis of WTAP and METTL3 expression after LPS+ATP and stretch stimulation. H IF analysis of WTAP expression in LPS+ATP-induced and stretch-induced primal cortex neuronal injury, scale bars =200 μm. Data are presented as the mean±SEM. *P<0.05, *versus* sham/control group.

As shown in Supplementary Figure S1, TBI caused neurological dysfunction in mice. The movement distance and speed of TBI mice were reduced, and they mainly stayed at the edge of the open field (Figure S1a). The mice spent more time and distance traveling to find the hidden platform in the maze, and the movement speed decreased (Figure S1b). Furthermore, Nissl and NeuN staining showed that TBI induced neuronal loss in the cerebral cortex tissue 48 h after TBI (Figure S2a and S2b). To investigate the significance of m6A in the mouse cerebral cortex after TBI, we assessed the levels of m6A regulators using qRT-PCR. The data found m6A ‘Writers’, ‘Erasers’, and ‘Readers’ were unbalanced, TBI induced WTAP, METTL3, and YTHYDF1 upregulation in a time-dependent manner and FTO was downregulated in mouse cerebral cortex (Fig. 1A). In contrast, the change in the WTAP was relatively large. The total changes in m6A levels in a time-dependent manner were significant, with 0.1422±0.0166% and 0.1446±0.0098% increase at 24 h and 48 h, respectively, in cerebral cortex after TBI (Fig. 1B). For the WTAP and METTL3 expression, WTAP was increased in a time-dependent manner in cerebral cortex after TBI but METTL3 expression did not change significantly by the WB analysis (Fig. 1C and D). The upregulation of WTAP mostly in the neurons was further confirmed by IF analysis at 48 h after TBI (Fig. 1E). Additionally, neuronal damage was induced by mechanical stretching and inflammation in vitro; 12% stretch stimulation and LPS + ATP treatment were used in subsequent experiments (Supplementary Figure S3a and 3b). In vitro primal neurons experiments showed that m6A ‘Writer’, ‘Eraser’, and ‘Readers’ were unbalanced (Fig. 1F), m6A level (Fig. 1G), with WTAP expression (Fig. 1H and I) being enhanced in LPS+ATP- and stretch-induced neuronal injury. These results demonstrated that m6A levels and WTAP expression of WTAP were upregulated in neuronal injuries after TBI.

### WTAP facilitates neuronal injury under stretch and TBI

**Figure 2:**
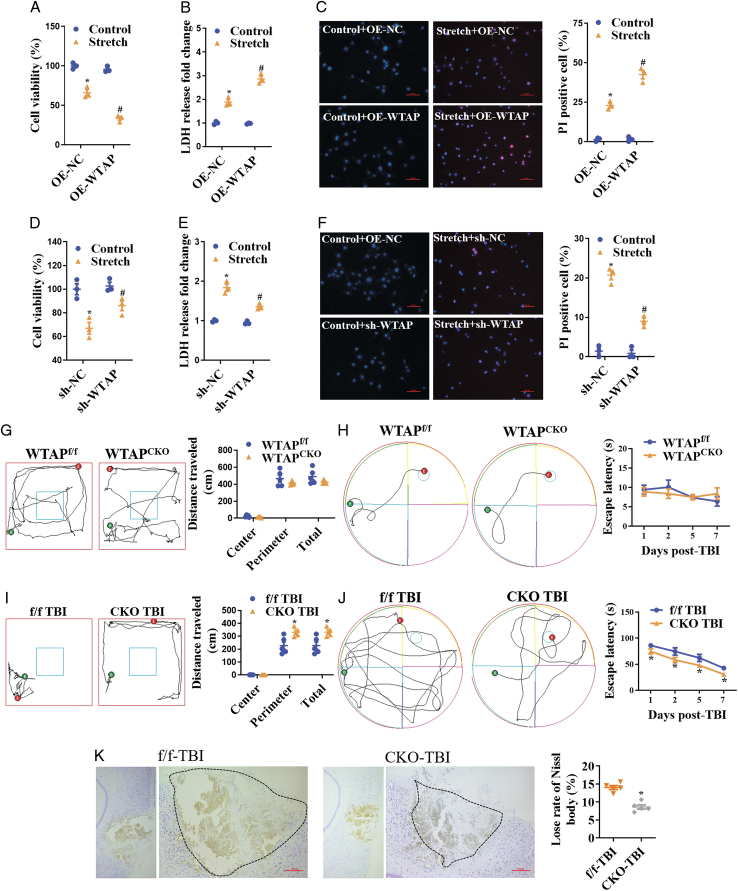
WTAP induced neuronal injury in vitro and WTAPCKO improved neurological dysfunction after TBI. Overexpression WTAP enhanced the increased neuronal injury after 12% stretch. (A) Detection of cell viability by the CCK8 assay. (B) Detection of LDH release. (C) Detection of positive PI (red) cells in stretch-induced primal cortex neurons transfected with WTAP overexpression plasmid. WTAP deletion reversed neuronal injury after 12% stretch. (D) Detection of cell viability by the CCK8 assay. (E) Detection of LDH release. (F) Detection of positive PI cells in stretch-induced primal cortex neurons transfected with sh-WTAP plasmid, scale bars =200 μm. Behavioral analysis was performed in neuronal conditioned knockout mice prior to trauma, including open field (G) and MWM (H). (I) Recording of the movement track of mice in open field at 48 h after TBI, including peripheral and central. (J) Interval track and escape latency (s) to find the platform during hidden platform trial of MWM. (K) Nissl stain analysis of neuronal loss at 48 h after TBI, scale bars =100 μm. Data are presented as the mean±SEM. *P<0.05, *versus* control/f/f-TBI group. #*P*<0.05, *versus* OE-NC/sh-NC group. OE, overexpression.

WTAP is an important regulator of m6A writers, then we assessed the function of WTAP in neuronal injury using WTAP overexpression lentiviral plasmid (sc-425635-LAC) or WTAP shRNA (m) lentiviral plasmid (sc-63225-V) and the transfection efficiency was detected by qPCR and WB (Figure S4a). Stretch-induced neuronal injury was detected using CCK8 assay, LDH analysis, and propidium iodide (PI) staining in vitro. WTAP overexpression significantly aggravated stretch-induced neuronal injury, leading to decreased cell viability (Fig. 2A), increased lactate dehydrogenase (LDH) release (Fig. 2B), and positive PI staining (Fig. 2C). However, WTAP downregulation reversed stretch-induced neuronal injury (Fig. 2D-F). Cell viability was reduced to ~67% after 12% stretch stimulation but increased to ~86% in sh-WTAP-treated primal cortex neurons compared to the control group (Fig. 2D). LDH release increased 1.84-fold but was reduced to 1.36-fold in sh-WTAP-treated primal cortex neurons compared to the control group (Fig. 2E). Furthermore, PI-positive staining was significantly reduced in the stretched sh-WTAP group compared to the stretched sh-NC group (Fig. 2F). These results suggested that WTAP positively regulates neuronal injury during stretching.

To further confirm the role of WTAP in neuronal damage, we constructed a TBI model using neuronal WTAP^[flox/flox, Camk2a-cre]^ (WTAP^CKO^) and WTAP^f/f^ mice and detected neuronal damage. There were no differences in behavioral function between WTAP^CKO^ and WTAP^f/f^ mice (Fig. 2G and H). In the open-field task, WTAP^f/f^ TBI mice spent less time in the perimeter zone and traveled a shorter total distance than WTAP^CKO^ TBI mice (Fig. 2I), but there was no difference in speed (Figure S1c). In the water maze task, spatial memory was analyzed. TBI induced a long latency time for spatial memory, but WTAP^CKO^ mice presented less latency time and movement distance (Fig. 2J and S1d). WTAP^CKO^ mice presented a longer quadrant time (%) in the target object compared to the WTAP^f/f^ mice following TBI, which implied that WTAP^CKO^ mice had a better memory after TBI (Fig. 2K); however, the speed was not different between WTAP^f/f^ and WTAP^CKO^ TBI mice (Figure S1d). TBI increased Nissl body loss, but decreased it in WTAP^CKO^ TBI mice, which showed a small area of damage in WTAP^CKO^ TBI mice (Fig. 2K).

### WTAP/m6A induces NLRP3 inflammasome after neuron injury

**Figure 3:**
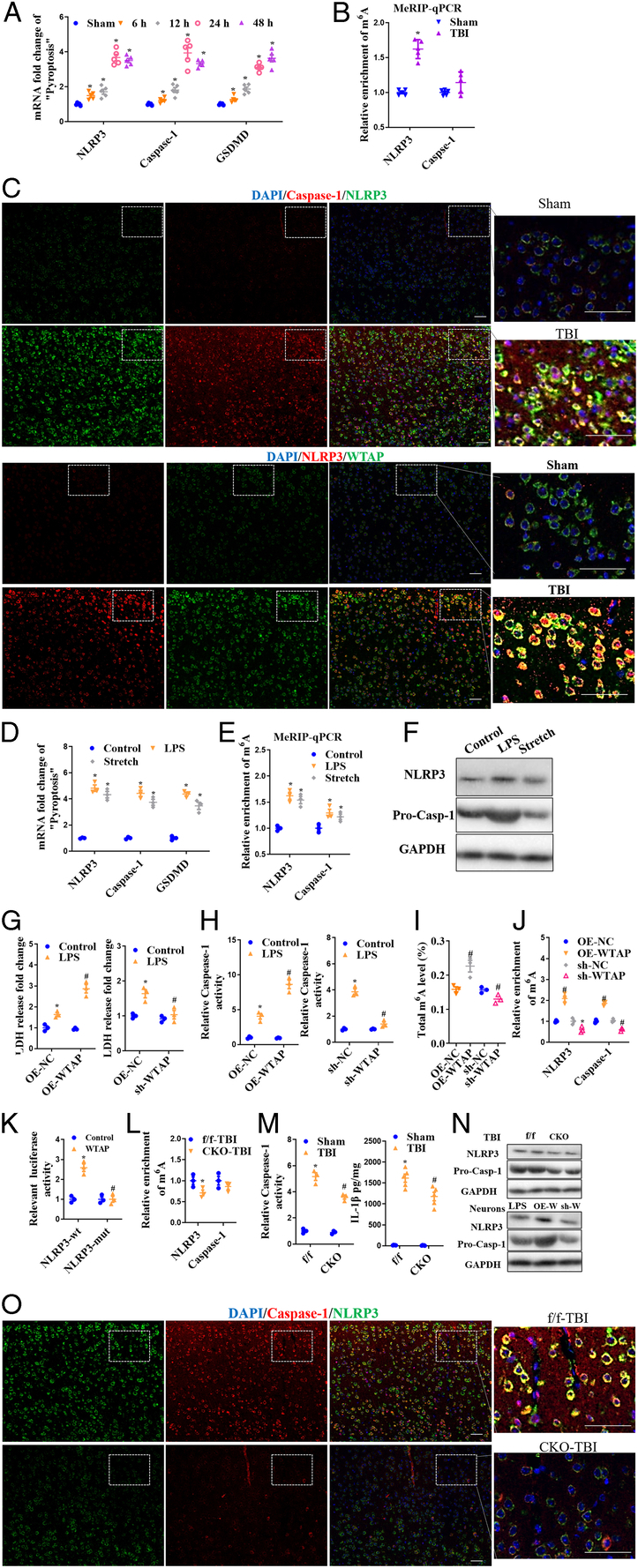
Increased NLRP3 m6A modification after nerve injury and WTAP/m6A regulated NLRP3 inflammasome after neuron injury. After TBI, (A) qRT-PCR analysis of NLRP3, Caspase-1 and GSDMD; (B) MeRIP-qPCR analysis of NLRP3 and Caspase-1 m6A levels at 12 h after TBI; (C) WB analysis of NLRP3 and Caspase-1 protein. After LPS and mechanical stretching induced cortical neuron injury, (D) qRT-PCR analysis of NLRP3, Caspase-1 and GSDMD mRNA levels; (E) MeRIP-qPCR analysis of NLRP3 and Caspase-1 m6A levels; (F) WB analysis of NLRP3 and Caspase-1 protein expression. LDH release levels (G), Caspase-1 activity (H), and IL-1β level (I) were detected in neurons with WTAP overexpression or WTAP down-regulation in LPS+ATP induced primary cortical neuron injury models. m6A levels were detected after LPS+ATP induced injury in neurons upregulated or downregulated by WTAP (J), and m6A levels of NLRP3 and Caspase-1 were detected by MeRIP-PCR (K). (L) The mutation of m6A motif of NLRP3 ‘A’ to ‘T’, and the luciferase reporting analysis of the binding of WTAP and NLRP3 in HEK293T cells. Detection of NLRP3 and Caspase-1 m6A levels of WTAP-CKO mice (M), and Caspase-1 activity and IL-1β level (N) in cerebral cortex at 48 h after TBI. (O) WB analysis of NLRP3 and Caspase-1 protein expressions in vivo and in vitro. (P) IF analysis of NLRP3 and Caspase-1 expression in cerebral cortex after TBI. OE-W, overexpression WTAP. Data are presented as the mean±SEM. *P<0.05, *versus* control/sham/NLRP3-wt group; #*P*<0.05, *versus* NC/f/f-TBI group. Scale bars =50 μm.

Following neuronal damage, NLRP3 inflammasome was detected. In the in vivo TBI model, NLRP3, Caspase-1, GSDMD mRAN were analyzed at 6, 12, 24, and 24 h and were all increased in the cerebral cortex after TBI (Fig. 3A). MeRIP-qPCR was used to analyze NLRP3 and Caspase-1 m6A levels 12 h after TBI and showed that NLRP3 m6A was significantly upregulated (Fig. 3B). IF analysis showed NLRP3 and Caspase-1expression was increased, and the co-stain of NLRP3/Caspase-1 and NLRP3/WTAP was stimulated 24 h after TBI (Fig. 3C). Furthermore, in vitro LPS and mechanical stretching induced cortical neuron injury model, we also detected increase of NLRP3-related molecule mRNA (Fig. 3D) and protein expression (Fig. 3F), as well as significant upregulation of NLRP3 m6A (Fig. 3E). These data suggest that NLRP3 m6A levels may be related to NLRP3 expression and NLRP3 inflammasome activation. Therefore, we investigated the effects of WTAP on NLRP3 expression. In LPS+ATP induced neuronal damage, overexpression of WTAP further aggravated LDH release and Caspase-1 activation, but downregulation of WTAP significantly alleviated LDH release, Caspase-1 activation, and IL-1β level (Fig. 3G-I). WTAP also regulated total m6A levels (Fig. 3J), and NLRP3 and Caspase-1 m6A levels (Fig. 3K), which were positively correlated with WTAP expression levels. When the m6A motif of NLRP3 ‘A’ was mutated to ‘T’, the luciferase reporting analysis demonstrated that WTAP could bling to NLRP3 (Fig. 3L), and WTAP^CKO^ decreased NLRP3 m6A level but not Caspase-1 m6A level in cerebral cortex (Fig. 3M), showing that WTAP mediated NLRP3 m6A modification depends on the m6A motif of NLRP3. Caspase-1 activity and IL-1β levels were measured in cerebral cortex at 48 h after TBI. WTAP^CKO^ mice exhibited significantly lower Caspase-1 activity and IL-1β levels than WTAP^f/f^ mice after TBI (Fig. 3N). WTAP downregulation not only reduced NLRP3 protein expression in TBI mice, but also attenuated LPS + ATP-induced primary neuronal damage (Fig. 3O). IF analysis of NLRP3/Caspase-1 expression in the cerebral cortex after TBI further confirmed these findings (Fig. 3P).

### WTAP promotes NLRP3 protein translation after neuronal injury in an m6A-YTHDF1 dependent manner

**Figure 4:**
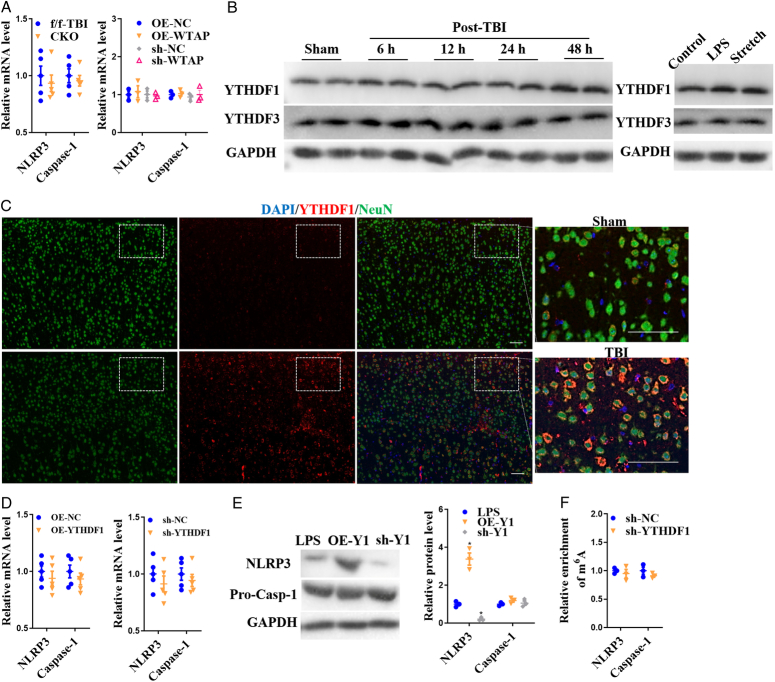
YTHDF1 mediated NLRP3 protein expression after neuronal injury. (A) Detection of NLRP3 and Caspase-1 mRNA levels in WTAP^CKO^ TBI mice and LPS+ATP-induced primal cortex neurons. (B) WB analysis of YTHDF1 and YTHDF3 expression after TBI and neuron injury. (C) IF analysis of YTHDF1 and NLRP3 expression in cerebral cortex after TBI. (D) mRNA levels of NLRP3 and Caspase-1 and (E) expression of NLRP3 and Caspase-1 protein after LPS-induced neuron injury in YTHDF1 upregulated or downregulated neurons. (F) The NLRP3 and Caspase-1 m6A levels were not down-regulated by YTHDF1 after LPS-induced neuronal injury. Y1: YTHDF1. Data are presented as the mean±SEM. *P<0.05, *versus* LPS+ATP group. Scale bars =50 μm.

Interestingly, downregulation of WTAP significantly regulated NLRP3 protein expression but did not affect NLRP3 and Caspase-1 mRNA levels, which were demonstrated in in vivo WTAP^CKO^ TBI mice and LPS+ATP-induced primary WTAP overexpression or deletion neuronal damage models (Fig. 4A). We suspected that WTAP/m6A mediated NLRP3 expression may be involved in the regulation of protein translation. We examined the protein levels of m6A ‘readers’ YTHDF1 and YTHDF3 (following the analysis of mRNA expression of YTHDF1 and YTHDF3, in Fig. 1A). YTHDF1 expression was upregulated in both TBI and neuron damage model, while YTHDF3 expression did not exhibit significant changes (Fig. 4B and C). Furthermore, neurons were transfected with sh-YTHDF1 or OE-YTHDF1 (Figure S4b). Interestingly, YTHDF1 did not significantly affect the gene expression (Fig. 4D) or the m6A levels (Fig. 4E) of NLRP3 and Caspase-1; however, it did exert a significant regulatory effect on NLRP3 protein expression. Specifically, sh-YTHDF1 decreased NLRP3 expression while the overexpression of YTHDF1 increased NLRP3 expression) (Fig. 4F). This suggests that the m6A reader, YTHDF1, mediates NLRP3 expression after neuronal injury.

**Figure 5:**
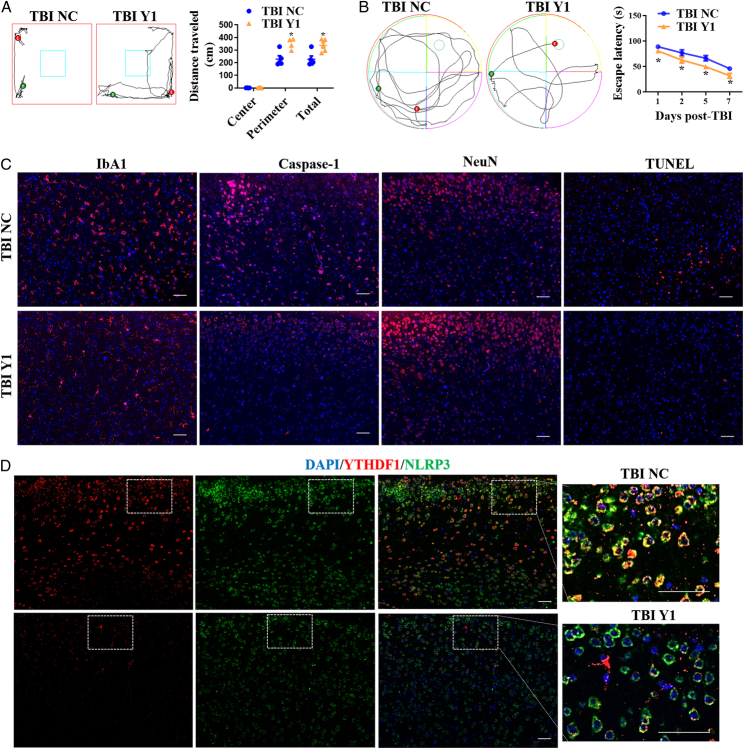
YTHDF1 KD improved TBI-induced nerve injury and NLRP3 expression. A YTHDF1 KD mice were generated by intraventricular injection of pAAV-U6-shRNA-YTHDF1 and were subjected to brain damage. Open field (A) and MWM (B) were used to analyze behavior. (C) IF analysis of IbA1 and caspase-1 expression in the cerebral cortex, neuronal loss by NeuN staining, and cell damage by TUNEL staining 24 h after TBI. (D) YTHDF1/NLRP3 co-staining in the cerebral cortex 24 h after TBI. scale bars =50 μm. Data are presented as the mean±SEM. *P<0.05, versus TBI-NC group. Y1, YTHDF1.

To reveal the role of YTHDF1 in vivo, YTHDF1 KD mice were made via intraventricular injection of pAAV-U6-shRNA-YTHDF1 and were subjected to brain damage. Behavioral analysis and expression levels of related molecules were subsequently detected. YTHDF1 downregulated mice showed better behavioral function, traveled through the central region than control mice (Fig. 5A), and presented better memory ability after TBI than control mice (Fig. 5B). YTHDF1 KD in mice resulted in inhibited IbA1 and caspase-1 expression, while NeuN staining intensity increased in the cerebral cortex following TBI. Additionally, TUNEL staining revealed reduced tissue damage after TBI in the YTHDF1 KD mice (Fig. 5C). YTHDF1/NLRP3 co-staining demonstrated that TBI induced the co-expression of YTHDF1 and NLRP3, and that YTHDF1 downregulation significantly decreased NLRP3 expression in the cerebral cortex after TBI (Fig. 5D).

**Figure 6:**
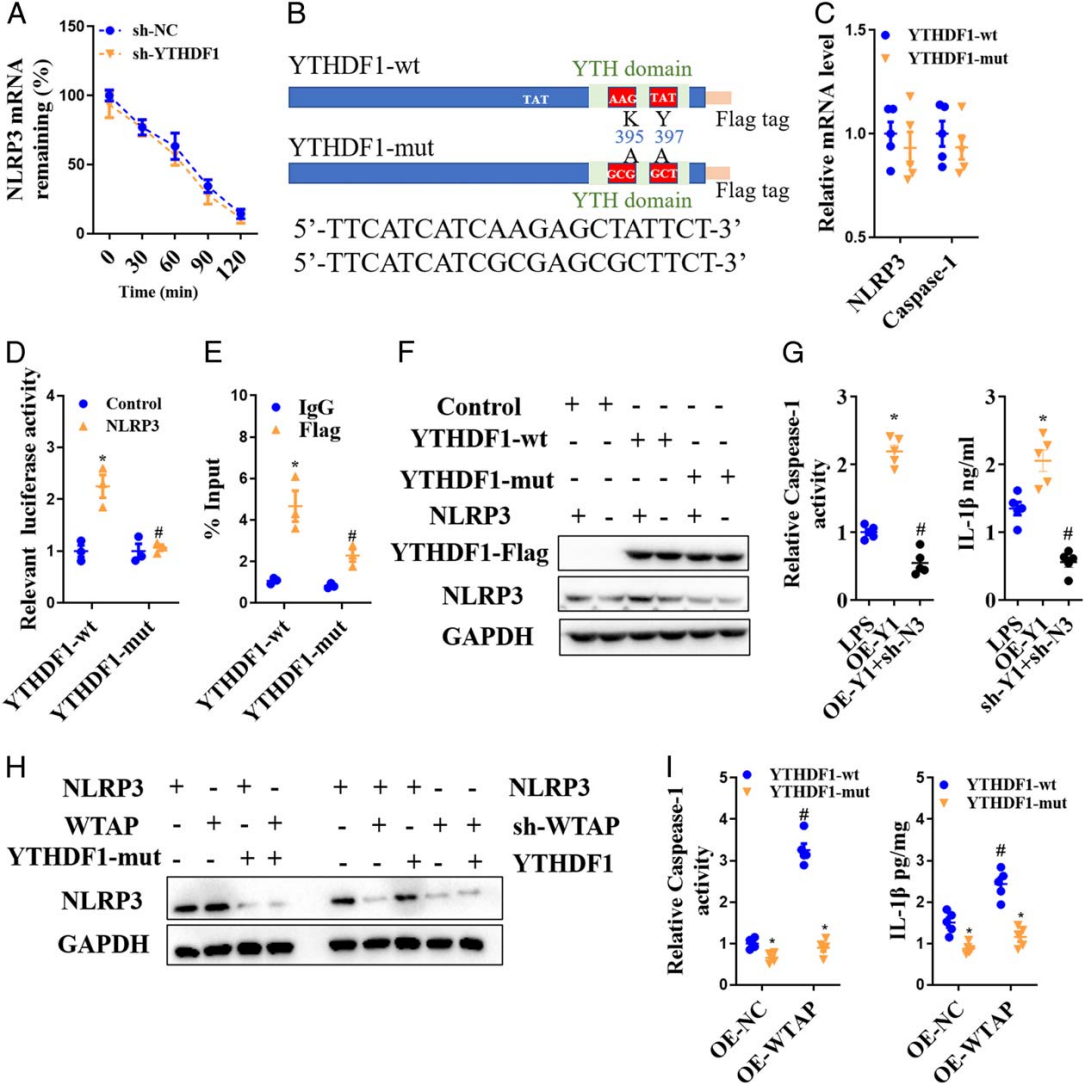
YTHDF1 regulates NLRP3 protein translation. (A) After actinomycin D treatment, mRNA degradation levels in YTHDF1 downregulated primary cortical neurons were detected. (B) YTH domain mutation design of YTHDF1. (C) After mutation in the YTH domain of YTHDF1, neuronal damage was induced by LPS+ATP and NLRP3 and Caspase-1 mRNA levels were detected. (D) The luciferase reporting analysis of the binding of YTHDF1 and NLRP3 in HEK293T cells. Binding of YTHDF1-wt or YTHDF1-mut to NLRP3 transcripts was detected by RNA-IP-qPCR analysis in primary neurons (E). WB analysis of the effect of YTHDF1 mutation on NLRP3 protein expression (F); analysis of the effect of YTHDF1 on Caspase-1 activity and IL-1β release (G); WB analysis of NLRP3 in WTAP overexpression and YTHDF1 mutant (H); ELISA analysis of Caspase-1 activity and IL-1β release in WTAP overexpression and YTHDF1 mutant (I). Data are presented as the mean±SEM. d and e, *P<0.05, *versus* control/IgG group; #*P*<0.05, *versus* YTHDF1-wt group. g, *P<0.05, *versus* LPS+ATP group; #*P*<0.05, *versus* sh-YTHDF1 group. i, *P<0.05, *versus* YTHDF1-wt group; #*P*<0.05, *versus* OE (overexpression)-NC group. Scale bars =50 μm.

After treatment with the transcription inhibitor actinomycin D, mRNA degradation levels in YTHDF1-downregulated primary cortical neurons were detected, while NLRP3 mRNA levels remained unchanged between the sh-NC and sh-YTHDF1 groups (Fig. 6A). As shown in YTH domain mutation design of YTHDF1 (Fig. 6B), the YTHDF1 mutant (YTHDF1-mut) was labeled with two key amino acid mutations (K395A and Y397A) to eliminate its m6A binding pocket. After the YTHDF1 mutation, NLRP3 and Caspase-1 mRNA levels were not affected by LPS + ATP-induced neuronal injury (Fig. 6C). Luciferase reporter assay and RNA-IP-qPCR analysis demonstrated that the YTHDF1 mutant decreased the binding of YTHDF1 and NLRP3 (Fig. 6D and E). In HT22 cells, m6A reader YTHDF1 mutant suppressed NLRP3 protein expression, while NLRP3 gene overexpression did not reverse the NLRP3 protein expression in YTHDF1 mutant neurons (Fig. 6F). Meanwhile, YTHDF1 overexpression also significantly aggravated LPS+ATP-induced Caspase-1 activation and IL-1β release but were reversed by the NLRP3 downregulation (Fig. 6G). Furthermore, we also revealed the role of YTHDF1 in WTAP-mediated NLRP3 expression in HT22 cells after LPS+ATP treatment (Fig. 6H). WTAP overexpression promoted NLRP3 protein expression (Fig. 6H), as well as Caspase-1 activity and IL-1β release (Fig. 6I), but the promotion effect was also repressed after YTHDF1 mutation. Thus, YTHDF1 is likely involved in the WTAP/m6A-mediated translation of NLRP3 protein following neuronal injury.

## Discussion

**Figure 7:**
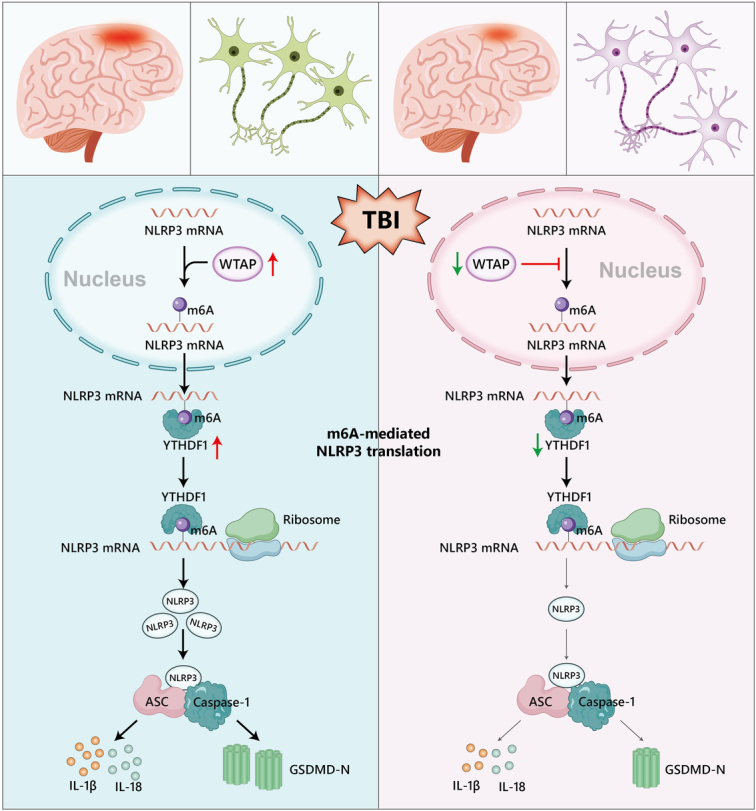
The preliminary mechanism of WTAP, which regulates NLRP3 m6A modification and promotes NLRP3 protein expression through the recognition of NLRP3 m6A modification by YTHDF1 m6A reader protein, ultimately regulating NLRP3 inflammasome activity after TBI.

m6A is a class of RNA modifications prevalent in the eukaryotic brain that is widely involved in biological functions and affects disease progression. The heterogeneity of RNA m6A modifications has been discussed in the mouse hippocampus and rat cortex^24,25^; however, no data on RNA m6A methylation in the cortex of commonly used TBI mouse models, hence, m6A function in TBI remain to be revealed. To our knowledge, this is the first study to reveal m6A modifications in the cerebral cortex following TBI in mice. We found that WTAP was positively correlated with NLRP3 inflammasome activation after TBI and mechanical stretch-induced neuronal injury. WTAP CKO in neurons alleviated neuronal injury and neurological dysfunction after TBI, and WTAP silencing reduced stretch-induced neuronal injury by inhibiting the m6A modification of NLRP3-mediated NLRP3 inflammasome activity. Interestingly, WTAP affected NLRP3 m6A modification levels and NLRP3 protein expression but did not control NLRP3 mRNA levels, suggesting that the regulation of NLRP3 expression by WTAP through m6A modification may be related to protein translation. Further analysis revealed that the YTHDF1 m6A reader protein recognized the m6A modification of NLRP3 and regulated NLRP3 protein expression and that YTHDF1 silencing improved stretch-induced neuronal injury by suppressing NLRP3 expression. Notably, we demonstrated the preliminary mechanism of action of WTAP, which regulates NLRP3 m6A modification and promotes NLRP3 protein translation through the recognition of NLRP3 m6A modification by the YTHDF1 m6A reader protein (Fig. 7), ultimately regulating NLRP3 inflammasome activity after TBI. Currently, WTAP physiological functions of WTAP in the brain are still not yet well understood, and further research is needed to construct a more complete understanding.

Previous studies have shown that m6A plays vital roles in neurodegeneration and inflammation. m6A modification regulates the inflammatory response in microglia and methylates mRNA and lncRNA in proinflammatory microglia 1-like/ unstimulated microglia 0-like (M0-L) involved in the immune system, signal transduction, and protein degradation processes. m6A modification also regulates methylated mRNA in anti-inflammatory microglia 2-like/M0-L involved in genetic information processing, metabolism, cellular processes, and neurodegenerative disease-related pathways^39^. ALKBH5 deletion enhances IFN-γ and CXCL2 mRNA m6A levels, thereby reducing the mRNA stability and protein expression in CD4+T cells, which has a protective effect on experimental autoimmune encephalomyelitis^40^. Du *et al*.^41^ have found that APOE ɛ4 is associated with m6A ‘writers’ METTL3, METTL16, YTHDC2, RBMX, and LRPPRC in the AD brain, and APOE ɛ4^+^ AD mice present high levels of METTL3^42^. Zhao *et al*.^43^ have demonstrated that METTL3 decrease-mediated m6A dysregulation contributes to neurodegeneration and may be a therapeutic target for patients with AD. The level of m6A and METTL3 expression in hippocampus and cortex neurons of AD patient are downregulated. Furthermore, METTL3 knockout reduces m6A modification in hippocampus and results in memory loss, neurodegeneration, spine loss, gliosis, oxidative stress, and aberrant cell cycle events, while METTL3 overexpression saves Aβ-caused synaptic injury and cognition disorders^43^. FTO upregulation decreases poststroke m6A hypermethylation, and exogenous FTO substantially decreases poststroke gray and white matter injury and improves motor function recovery, cognition, and depression-like behavior^44^. The mRNA m6A levels and ALKBH5 expression are upregulated in MCAO rats and OGD-induced primary neurons, while FTO expression is reduced. Furthermore, ALKBH5 inhibition exacerbates neuronal damage via ALKBH5/FTO co-regulation of BCL2 demethylation, BCL2 transcript degradation, and Bcl2 expression^21^. In vivo, upregulated FTO reduces cerebral ischemia infarction and cell death, which mediates Nrf1 mRNA m6A levels to increase Nrf2 expression, leading to oxidative stress inhibition and improvement of cerebral I/R damage^45^. YTHDF1 is increased in oxygen-poor conditions, and YTHDF1 silencing decelerates the infarct area and neuronal injury in I/R by inducing PTEN degradation^46^. The FTO inhibitor MO-I-500 inhibits streptozotocin-induced oxidative stress and ameliorates astrocyte survival, GFAP expression, mitochondrial dysfunction, and bioenergy disturbances^47^. Downregulated METTL14/m6A inhibits neuronal death following spinal cord injury^48^. In diabetic mice, the expression of YTHDF1, YTHDF3, and WTAP is significantly downregulated in the hippocampus, while increasing the hippocampal YTHDF1 improve STZ-induced diabetic cognitive dysfunction^49^. Therefore, the targeting of m6A modifications has become a possible treatment option for nervous system-related diseases. Furthermore, m6A is associated with the pathological progression after TBI; however, its specific role and mechanism have not been reported^24,25^. In this study, we explored the epigenetic methylation of RNA and m6A and found that WTAP expression was upregulated in the mouse cerebral cortex following TBI and neuronal damage in vitro. It was further confirmed via conditional knockdown of neurons that WTAP CKO ameliorated neuronal injury, neuroinflammation, and neurological dysfunction after TBI, and that WTAP shRNA inhibited in vitro neuronal damage by decreasing m6A levels. These data suggest that the overexpression of WTAP impairs neuronal survival post-TBI through m6A modifications, and targeting WTAP may be a potential therapeutic approach.

WTAP, a vital component of the m6A writer, has been shown to contribute to the progression of various cancers, stabilizing the expression of the transcriptional repressor BCL6 in diffuse large B-cell lymphoma^50^, promoting the progression of hepatocellular carcinoma via ETS1 silence^51^, influencing tumor-associated T lymphocyte infiltration in gastric cancer^52^, and predicting poor prognosis in acute myeloid leukemia by regulating MYC mRNA^53^. Extensive studies have revealed the biological functions of WTAP. WTAP affects the expression and activity of human lactonase PON2 via post-transcriptional mechanisms^54^. WTAP contributes to the pathogenesis of psoriasis by promoting keratinocyte proliferation via the induction of cyclinA2-mediated and CDK2-mediated cell cycle progression^55^. In arteriovenous malformations of the brain, WTAP/m6A regulates endothelial cell angiogenesis via β-catenin degradation^56^. WTAP presents a vital role in regulating carbonic anhydrase IV on Wnt/β-catenin pathway. WTAP KD abolishes the role of acetazolamide (AZA) in β-catenin expression, indicating WTAP involvement in the protective effects of AZA on homocysteine-caused blood-brain-barrier disruption by mediating the Wnt/β-catenin pathway^57^. Recently, it was reported that WTAP promotes endoplasmic reticulum stress by regulating ATF4 mRNA m6A levels in myocardial cells^58^. Astragalus mongholicus polysaccharides (APS) reduces LPS-induced IL-6 levels, m6A modification levels, and WTAP gene expression in THP-1 macrophages, and overexpression of WTAP reverses APS-induced IL-6 expression via WTAP-mediated p65 nuclear translocation^59^. Lan *et al*.^60^ demonstrated that WTAP expression is increased in patients with diabetic nephropathy and in high-glucose (HG)-treated HK-2 cells, and is positively associated with NLRP3 inflammasome components and proinflammatory cytokines. WTAP regulated NLRP3 expression, whereas WTAP-KD attenuated HG-induced pyroptosis and NLRP3-related proinflammatory cytokines in both HK-2 cells and diabetic mice^60^.

The NLRP3 inflammasome is considered as a potential biomarker and therapeutic target in the pathological process of TBI^28,29^. Therefore, much research has been devoted to the study of endogenous inhibitors or activators of the NLRP3 inflammasome, with the aim of deeply exploring the NLRP3 inflammasome regulatory network^38,61–65^, and it has been identified as a key molecule in the regulation of inflammation and immunity^66^. m6A modification sites are both found in mouse and human NLRP3 genes. Schwartz *et al*.^67^ have found that LPS stimulates the m6A peak of NLRP3 in mouse dendritic cell, which suggests that NLRP3 expression and inflammasome activation maybe mediated by m6A modification.

m6A regulators mediate NLRP3 expression under various pathological conditions. In an in vivo atherosclerosis model, partial ligation of the carotid artery led to plaque formation and upregulation of METTL3, while METTL3 silencing prevented NLRP3 upregulation, KLF4 downregulation, and atherogenic processes^33^. Furthermore, METTL14/mRNA mediates NLRP3 mRNA stability in arsenic-induced hepatic insulin resistance^34^. METTL3 expression is elevated in MI/R rats and OGD/R cardiomyocytes and aggravates cardiomyocyte pyroptosis and MI/R damage, whereas METTL3 silencing alleviates myocardial injury by suppressing NLRP3 inflammasome activation^35^. Zhang *et al*.^36^ demonstrated that the m6A reader protein YTHDF1 alleviates NLRP3 inflammasome-mediated sepsis by increasing WWP1 translation in CLP-induced mice and LPS + ATP-induced RAW264.7 cells. However, another study shows the opposite result that YTHDF1 induces proinflammatory IL-1β production in macrophages following bacterial infection. Upregulated YTHDF1 causes NLRP3 translation, and YTHDF1 deficiency promotes survival of sepsis mice^68^. We also verified that YTHDF1 levels were positively correlated with NLRP3 inflammasome components and proinflammatory cytokines after TBI and in vitro neuronal injury. Our findings demonstrate that the YTH domain of YTHDF1 could directly bind to NLRP3 mRNA. This interaction, mediated by YTHDF1 m6A reader protein recognition of the m6A modification of NLRP3, regulates NLRP3 protein expression. Furthermore, YTHDF1 mutation or silence not only improved neuronal injury but also inhibited Caspase-1 activation and IL-1β levels. This protective effect is likely due to the suppression of NLRP3 protein translation, effectively reversing the stimulatory effect of WTAP overexpression on NLRP3 expression and inflammation.

Unlike some previous reports, where m6A was shown to regulate NLRP3 mRNA stability or expression via indirect role^36,69^, we revealed for the first time that WTAP directly acts on NLRP3 mRNA and regulates its m6A modification, and that the YTH region of YTHDF1 plays a key role in regulating the translation of NLRP3 protein and WTAP-mediated NLRP3 in neuronal injury. However, the m6A modification and regulatory network of NLRP3 may have more complex and changeable pathological mechanisms in different experimental models and processing nodes. Relevant studies need to be more detailed in order to obtain more valuable theoretical support.

## Conclusions

We investigated the function of WTAP/YTHDF1 in neuronal injury following TBI. Mechanistically, WTAP acted directly on NLRP3 mRNA, regulated NLRP3 mRNA m6A levels, and promoted NLRP3 expression following neuronal injury. Further analysis found that YTHDF1 directly mediated NLRP3 protein translation and the inflammatory response, and downregulation of WTAP/YTHDF1 improved neuronal injury and neurological dysfunction after TBI. Notably, we found that WTAP/m6A/YTHDF1-mediated NLRP3 protein translation promotes the pathological progression of TBI, and downregulation of WTAP/m6A /YTHDF1 is a viable and promising therapeutic approach for preserving neuronal function after TBI, which can provide support for targeted drug development.

## Ethical approval

Ethical approval for this study was approved by the Animal Care and Use Committee of Xi’an Peihua University, China, on 10 March 2019, in accordance with the ARRIVE guidelines.

## Consent

Not applicable.

## Source of funding

This study was supported by the National Natural Science Foundation of China (No. 82072229, 81901270, 82172196, 82202440, and 82372507), Science and Technology Support Plan of Guizhou Province ([2023] general 088), Natural Science Basic Research Program of Shaanxi (No. 2023-JC-QN-0932 and 2024SF-YBXM-217), Special Research Project of Traditional Chinese Medicine Science and Technology of Guizhou (QZYY-2023-022 and QZYY-2023-025), and Basic Research Programme of Guizhou Province ([2024] general 586).

## Author contribution

Y.H.C., T.L.L., J.H.C., K.X., and C.Z.: planned and designed the research; Y.H.C., J.H.C., J.M., T.L.L., J.N.W., H.W., M.L.K., and X.Z.: performed animal experiments and cell experiments; J.H.C., J.M., and Q.H.X.: provide the statistical analysis; Y.H.C.: wrote the original draft; Q.H.X. and C.Z.: contributed to review and editing; Y.H.C., T.L.L., H.W., K.X., and C.Z.: conducted the funding acquisition; K.X.: performed the project administration and supervision. All authors read and approved the final manuscript.

## Conflicts of interest disclosure

The authors declare that they have no competing interests.

## Research registration unique identifying number (UIN)

1. Name of the registry: not applicable.
2. Unique identifying number or registration ID: not applicable.
3. Hyperlink to your specific registration (must be publicly accessible and will be checked): not applicable.

## Guarantor

Chi Zhang.

## Data availability statement

All datasets used and/or analyzed in this article are stored on the data server of the corresponding authors’ lab and can be accessed by E-mail request to the corresponding authors.

## Provenance and peer review

Not applicable.

## Supplementary Material
