# Supplementary material for: WTAP participates in neuronal damage by protein translation of NLRP3 in an m6A-YTHDF1-dependent manner after traumatic brain injury
Source: Int J Surg. 2024 Jun 14;110(9):5396–408. doi: 10.1097/JS9.0000000000001794 (PMC11392096; doi:10.1097/JS9.0000000000001794)

**Supplementary materials**

**Animals and TBI models**

Ethical approval for this study was approved by the Animal Care and Use Committee of Xi’an Peihua University, China, in accordance with the ARRIVE guidelines.^37^ The floxed WTAP allele (WTAP^f/f^) C57BL/6J mice (purchased from Cyagen Biosciences Inc. (Guangzhou, China), then bred with neuronal conditional knockout (CKO) WTAP^[flox/flox, Camk2a-cre]^ mice using Camk2a-cre transgenic mice) and wild type (WT) mice (8 weeks, 20 ± 2 g) were exposed to a 12 h/12 h light-dark cycle, along with free activity and foraging (including food and water).

The animals were anesthetized with 2%~3% isoflurane inhalation anesthesia (RWD Life Science Co., Shenzhen, China; 1%~1.5% maintain) and maintained to a normal temperature of mice using a thermostatic heating pad at 37℃ during and after surgery. The mice were held in a stereotaxic frame and the scalp was exposed and cleaned with povidone-iodine. TBI was induced using a controlled cortical injury (CCI) device (RWD Life Science Co.) as described in our previous study.^32,38^

For YTHDF1 knockdown (KD) mice, YTHDF1 pAAV-U6-shRNA (targeting sequence: GCTGAAGATTATCGCTTCCTA; Beijing Syngenbio Co., Ltd., Beijing, China) and negative control (NC) pAAV-U6-shRNA were prepared according to the manufacturer’s instructions. The right lateral ventricles (depth: 3.0 mm) were injected with 2 μL 10^12^ VG/mL virus (targeting YTHDF1 or NC) at 1 μL /min; the needle remained in place for 2 min and was slowly recovered once the injection was complete. Follow-up experiments were conducted two weeks later.

Before the experiment, the behavior of all mice was consistent and normal, and none of the mice died during model construction. In total, 108 mice were used in this study. A total of 18 mice per group were categorized into WT sham, WT TBI, WTAP^f/f^ TBI, WTAP^CKO^ TBI, TBI YTHDF1 KD and TBI NC groups.

**Analysis of animal behavior**

Neurological deficits were assessed using the open-field and Morris water maze tests according to our previous studies.^32,38^ SMART 3.0 software (Panlab, Barcelona, Spain) was used to record and analyze the total travel distance and track the motion trial in the open-field assay.

Learning and memory functions of the mice were evaluated one week after TBI using the Morris water maze, as previously described. The circular pool was 120 cm in diameter and 50 cm deep, whereas the white platform was 6 cm in diameter and 30 cm high. An aqueous solution of titanium dioxide was injected into the pool approximately 1 cm above the platform. During the five-day training period, the platform was placed in the third quadrant of the test site. All mice were allowed 90 s to find the platform and 10 s to stay on it, or when no platform was found for 90 s, the mice were directed to a platform where they could rest for 10 s. In a daily timing trial, the mice were randomly placed in one of three quadrants (first, second, and fourth), with 4-minute intervals between tests. On the 6th day, each mouse was tested in one direction to assess cognitive function. SMART 3.0 software (Panlab, Barcelona, Spain) was used to record average latency, swimming speed, and swimming distance.

**Histopathological analysis**

After fixation with 4% paraformaldehyde, tissue paraffin 4 μm slices were conventionally dyed with hematoxylin and eosin (H&E) and Nissl staining to analyze the histopathological condition after TBI. For Nissl staining, the 4 μm sections were hydrated with 1% toluidine blue (Solarbio, Beijing, China) for 20 min at 50°C. After washing with double-distilled water, sections were dehydrated and mounted. For TUNEL assay, a DeadEnd™ fluorometric TUNEL system was used according to the manufacturer’s directions (Cat: G3250; Promega, Madison, WI, USA), followed by overnight incubation with primary antibodies anti-NeuN (Cat: ab177487; Abcam, 1:400). The nucleus was then stained with 4',6-diamidino-2-phenylindole (DAPI; Solarbio, Beijing, China). For IF staining, the sections were incubated overnight with primary antibodies anti-WTAP (Cat: 60188-1-AP; Proteintech Group, Inc., Wuhan, China; 1:200), anti-YTHDF1 (Cat: 12592-1-AP; Proteintech Group, Inc.), anti-NLRP3 (Cat: ab263899; Abcam, 1:400), Caspase-1 (Cat: ab177487; Abcam, 1:400), and anti-NeuN (Cat: ab177487; Abcam, 1:400), and the nucleus was stained with 4',6-diamidino-2-phenylindole (DAPI; Solarbio). The images were obtained using a fluorescence microscope (Leica, Oskar-Barnack, Germany).

**Cell culture and treatment**

Primary cortical neurons were prepared from the brains of 16-day-old C57BL/6 mouse embryos according to our preliminary research ,^30, 32^ and the cells were used for further experiments after culturing for eight days.

Neuronal damage can be induced by mechanical stretching and inflammation. For mechanical stress, neurons were inoculated in 6-well/24-well plates (BioFLEX®), and equiaxial stretch (12% strain, 1.0 Hz frequency) was applied to cultured neurons for 4 h using Flexcell® FX-5000™ Tension System (Flexcell, USA).^30^ For the inflammation-induced model, neurons were incubated for 4 h with 10 ng/ml LPS and 5 mM ATP for 30 min.

HEK293T and HT22 cells were cultured in DMEM medium (supplemented with 10% fetal bovine serum [FBS]) and were used for transfection and dual-luciferase reporter assays.

**Transfection**

Cells were transfected at 70%~80% confluence with shRNA or overexpression targeting WTAP, YTHDF1, or NLRP3 using Lipofectamine 3,000 (Invitrogen) for 48 h. WTAP overexpression lentiviral plasmid (sc-425635-LAC), WTAP shRNA (m) lentiviral plasmid (sc-63225-V), YTHDF1 overexpression lentiviral plasmid (sc-432834-LAC), YTHDF1 shRNA (m) lentiviral plasmid (sc-155423-V), NLRP3 overexpression lentiviral plasmid (sc-432122-LAC), NLRP3 shRNA (m) lentiviral plasmid (sc-45470-V), control lentiviral activation particles (sc-437282), control shRNA lentiviral particles (sc-108080) were purchased form Santa Cruz Biotechnology Inc. (Dallas, Texas, USA) and transfection efficiency was presented in Figure S4. FLAG-tagged YTHDF1-wt (5’-TTCATCATC**AAG**AGC**TAT**TCT-3’) and YTHDF1-mut (5’-TTCATCATC**GCG**AGC**GCT**TCT-3’) were structured by Sangon Biotech (Shanghai) Co., Ltd. (Shanghai, China).

**Dual-luciferase reporter assay**

The NLRP3 3′UTR sequence was cloned into a pGl3 vector (Promega, Madison, WI, USA). HEK293T cells were plated at 60%~70% confluence onto 24-well plates, cultured in DMEM supplemented with 10% FBS, and transduced with WTAP-, YTHDF1- shRNA, or overexpression vector and co-transfected with either the pGl3-NLRP3 3′UTR luciferase reporter plasmid or the pRL-TK vector (Promega) expressing the Renilla luciferase with Lipofectamine 3,000 (Invitrogen) for 48 h. Then, the luciferase activities were measured by the dual-luciferase reporter assay system (Promega).

**Global RNA m6A quantification**

The EpiQuik m6A RNA Methylation Quantification Kit (Colorimetric; Cat: P-9005; Epigentek Group Inc., Farmingdale, NY, USA) was used to assess the total m6A levels in cell or tissue RNAs. Briefly, 2 µL of NC, 2 µL of PC and 200 ng of RNA were added and mixed into strip well. m6A was assessed using capture and detection antibodies, and absorbance at 450 nm was detected using a microplate reader (Thermo Scientific, Wilmington, USA).

**mRNA stability measurements**

Primary cortical neurons were infected with sh-YTHDF1 or sh-NC. After 48 h transfection, actinomycin D was added into medium with a final concentration of 5 μg/mL, and cells were collected at 0, 30, 60, 90 and 120 min after treatment. Total RNA was extracted from primary cortical neurons, cDNA was synthesized, and the relative expression of NLRP3 mRNA was detected by qPCR.

**DAPI/PI fluorescent staining**

Primary cortical neurons were seeded in 24-well plates at a density of 12,000 cells/well. The medium of the treated cells was removed, and the cells were fixed with 4% paraformaldehyde for 10 min. For PI staining, 50 μg/ml PI solution were added and incubated at room temperature without light for 30 min. For DAPI staining, 0.5 μg/ml DAPI solution were added and incubated for 5 min at dark. Images were captured under a fluorescence microscope (Leica).

**LDH release detection**

Primary cortical neurons were plated in 96-well plates at a density of 6000 cells/well. After treatment, the LDH levels were measured using an LDH Cytotoxicity Assay Kit (Cat: C0016; Beyotime Biotechnology, Shanghai, China). Finally, the absorbance was measured at 490 nm using a microplate reader (Thermo Scientific).

**Caspase-1 activity analysis**

Cells or cerebral cortex tissues were harvested and lysed on ice for 10 min, centrifuged at 12,000×g at 4℃ for 10 min. Then, 50 μL lysate supernatant with 40 μL test buffer was mixed with 10 μL Ac-YVAD-*p*NA (2 mM) and incubated at 37°C for overnight. Finally, absorbance was measured at 405 nm using a microplate reader (Thermo Scientific).

**IL-1β detection**

Cerebral cortex tissues were lysed on ice for 10 min and centrifuged at 12,000×g at 4℃ for 10 min, and the supernatant of primary neurons was centrifuged at 1,000×g at 4℃ for 5 min. Then, IL-1β levels in cerebral cortex tissues and primary neurons were measured using a Mouse IL-1β ELISA Kit (Cat: PI301; Beyotime Biotechnology).

**RNA immunoprecipitation (RIP)**

The RNA immunoprecipitation assay was conducted using an RNA Immunoprecipitation (RIP) kit (Cat: Bes5101; BersinBio, Guangzhou, China). Briefly, 1 × 10^7^ neurons were lysed in the RIP lysis buffer. After removing DNA, 20 µL of protein A/G bead-conjugated anti-m6A (Cat: A-1801; 4 µg; Epigentek Group Inc., Farmingdale, NY), anti-YTHDF1 antibodies (Cat: 14392-1-AP; 3 µg; Proteintech Group, Inc.), or anti-IgG antibody were added to the samples and incubated overnight at 4°C. Subsequently, the precipitated beads were washed with RIP-wash buffer for 10 min at 4°C and then RIP-lysis buffer for 5 min at 4°C. The RNA in the immunoprecipitated complex and the input fraction were extracted by incubating cells at 65°C for 2 h with 200 mM NaCl and 20 μg proteinase K, which reversed the crosslinking. Finally, the coprecipitated RNAs was purified and analyzed by qRT-PCR.

**Western blot (WB) analysis**

Cells or cerebral cortex tissues were harvested and lysed on ice for 10 min and then centrifuged at 12,000×g for 10 min at 4℃. After centrifugation, the protein concentrations were determined using a BCA assay kit. Protein samples (30 µg) were fractionated on 12% SDS-PAGE gels. After Western blot transfer, the membranes were blocked with 5% BSA and incubated with the appropriate primary antibodies: METTL3 (Cat: 15073-1-AP; Proteintech; 1:2,000), anti-WTAP (Cat: 60188-1-AP; Proteintech; 1:1,000), anti-YTHDF1 (Cat: 12592-1-AP; Proteintech; 1:1,000), anti-YTHDF3 (Cat: 25537-1-AP; Proteintech; 1:1,000), anti-NLRP3 (Cat: ab263899; Abcam, 1:1,000), Caspase-1 (Cat: ab177487; Abcam, 1:800), anti-Flag (Cat:20543-1-AP; Proteintech; 1:1,000), and anti-GAPDH (Cat: 60004-1-Ig; Proteintech Group, Inc.; 1:200,000) at 4°C overnight, and then incubated with secondary antibodies (Abgent, 1: 20,000). The signal was detected using an ECL-Detection Kit (Millipore, USA). The full uncropped gel and blot images are shown in Figure S5.

**Quantitative RT–PCR**

Total RNA was extracted using the TRIzol reagent (Invitrogen, Carlsbad, CA, USA) according to the manufacturer’s instructions. The concentration and purity of all samples were measured using a NanoDrop 2000 (Thermo Scientific) and cDNA was synthesized using the Superscript First-Strand Complementary DNA Synthesis Kit (TIANGEN BIOTECH Co., Ltd., Beijing, China). Finally, the products were quantified using SYBR Green real-time PCR (TIANGEN BIOTECH Co.,LTD) on a Roche LightCycler 480II instrument. The nucleotide sequences of the primers were shown in Table S1.

**Statistical analysis**

Data are presented as means ± standard error of the mean (SEM). SPSS 21.0 (IBM, Armonk, NY, USA) was used to perform data analysis. For multiple comparisons of more than two groups and variates, data were analyzed using two-way ANOVA followed by Bonferroni’s post hoc test for normally distributed data or by the Kruskal-Wallis test for non-normally distributed data. All data examining groups over time were analyzed using repeated measures ANOVA, followed by Tukey’s post-hoc test to compare differences between groups at the same time. *P* < 0.05 was considered significant.

Table S1. Primers for RT-qPCR

|  | Primer | Sequence（5'-3'） |
| --- | --- | --- |
| Mettl3 | Forward primer | GGACTCTGGGCACTTGGATT |
|  | Reverse primer | GCACGGGACTATCACTACGG |
| Mettl14 | Forward primer | ATTGCAGCACCTCGGTCATT |
|  | Reverse primer | ACCCCACTTTCGCAAGCATA |
| Wtap | Forward primer | TGCAAGAGTGCACCACTCAA |
|  | Reverse primer | GTTGATCTCAGTTGGGCCAC |
| Fto | Forward primer | GTGTTTTGGCTGGCTCACAG |
|  | Reverse primer | GTCGCCATCGTCTGAGTCAT |
| Alkbh5 | Forward primer | GGGACCACCAAGCGGAAATA |
|  | Reverse primer | ATCCTGACTGAAGAGCCGGA |
| Ythdf1 | Forward primer | ACAGTTACCCCTCGATGAGTG |
|  | Reverse primer | GGTAGTGAGATACGGGATGGGA |
| Ythdf2 | Forward primer | AGCAGAGACCAAAAGGTCAAG |
|  | Reverse primer | CTGTGGGCTCAAGTAAGGTTC |
| Ythdf3 | Forward primer | CATAGGGCAACAGAGGAAACAG |
|  | Reverse primer | ATCTCCAGCCGTGGACCAT |
| Nlrp3 | Forward primer | GCTCCAACCATTCTCTGACC |
|  | Reverse primer | AAGTAAGGCCGGAATTCACC |
| caspase-1 | Forward primer | AGGAATTCTGGAGCTTCAATCAG |
|  | Reverse primer | TGGAAATGTGCCATCTTCTTT |
| Gapdh | Forward primer | AACTTTGGCATTGTGGAAGG |
|  | Reverse primer | GGATGCAGGGATGATGTTCT |

**Figure Legends:**

**
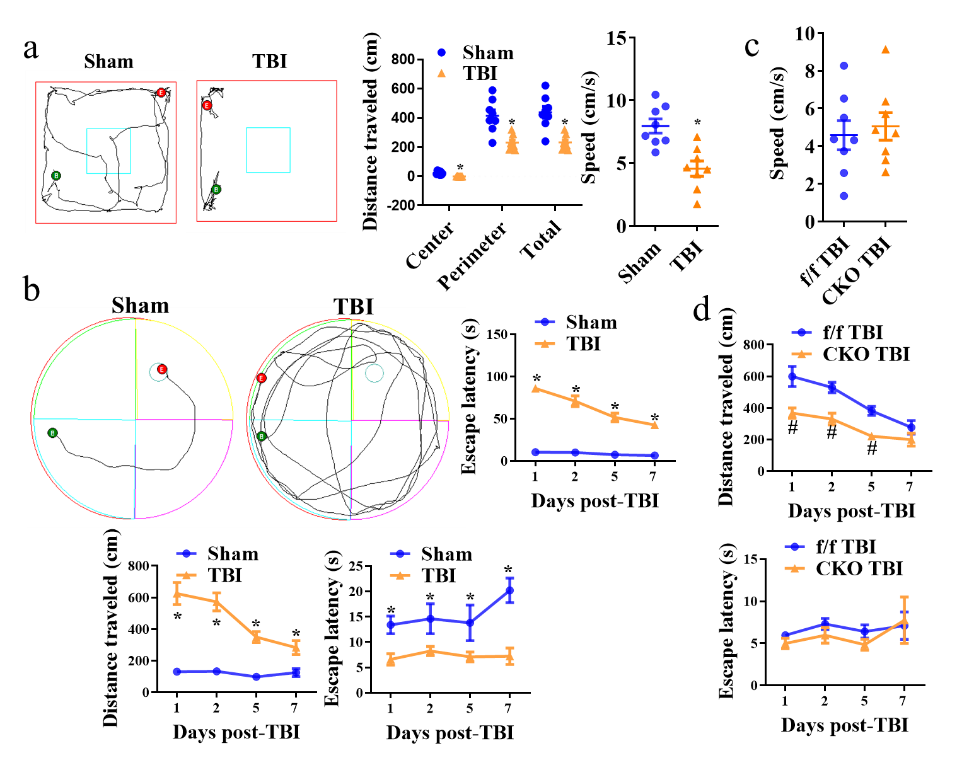
**

**Figure S1 Behavioral analysis of mice after TBI.** In WT mice, **(a)** the movement track of mice in open field was recorded at 48 h after TBI, including peripheral and central and speed of mice; **(b)** Hidden platform trial of MWM was conducted and recorded the interval track, escape latency (s), and speed to find the platform. In neuronal conditional knockout (CKO) WTAP^[flox/flox, Camk2a-cre]^ mice, **(c)** the speed of mice in open field was recorded at 48 h after TBI; (d) the travel distance and speed were recorded in the hidden platform trial of MWM. Data are presented as the mean ± SEM. **p* < 0.05, *vs.* sham group. #*p* < 0.05, *vs.* WTAP^f/f^ TBI mice.

**
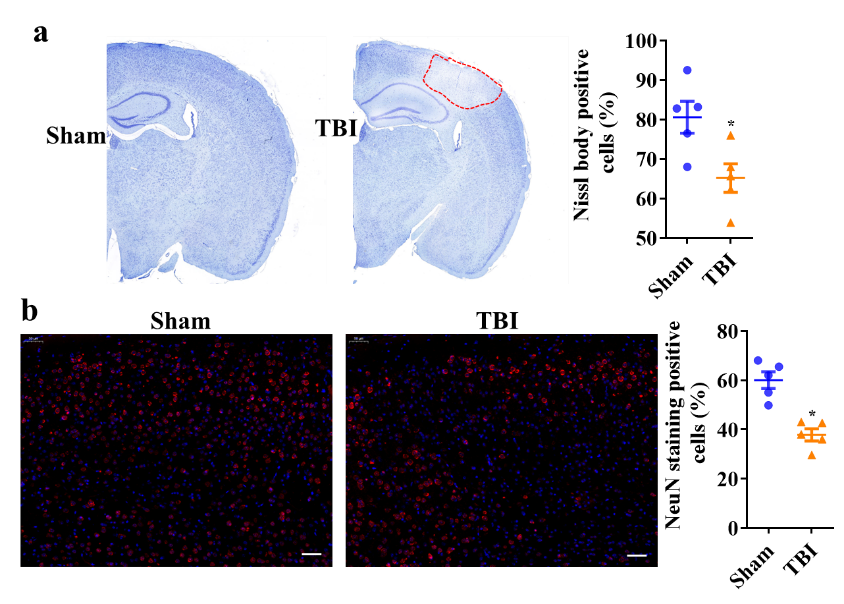
**

**Figure S2 TBI induced neuronal lose in** **cerebral cortex tissue. (a)** Nissl stain analyzed neuronal loss at 48 h after TBI, and scale bars. (b) IF analysis of NeuN in cerebral cortex at 48 h after TBI, and scale bars= 50 μm. Data are presented as the mean ± SEM. **p* < 0.05, *vs.* sham group.

**
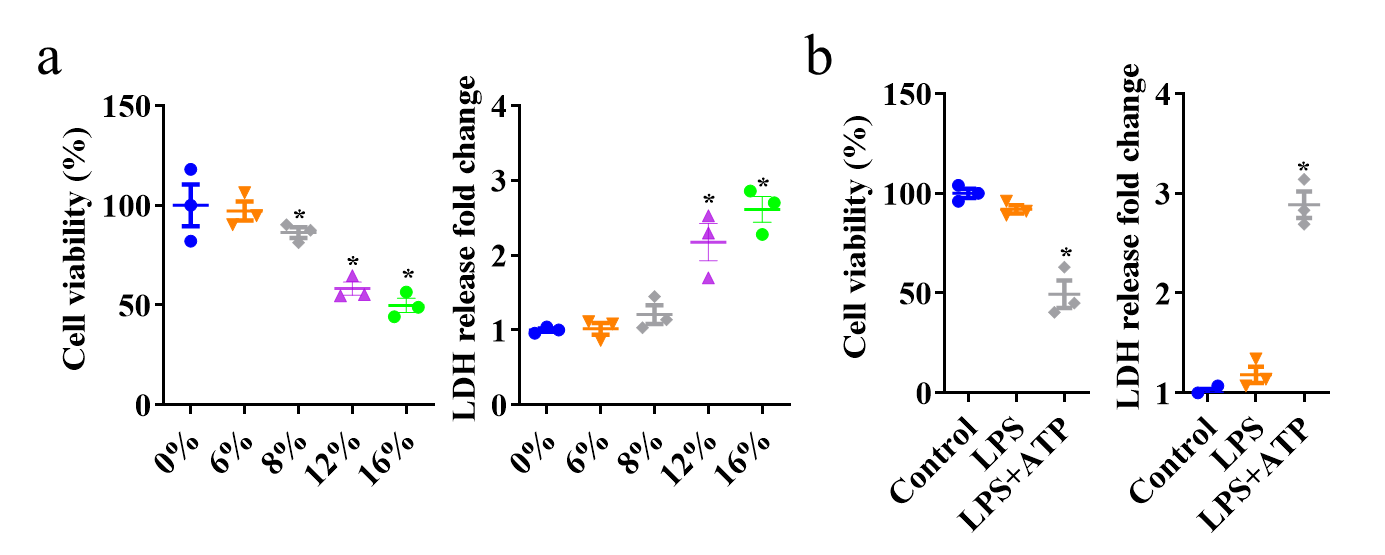
**

**Figure S3 Neuronal damage was induced by mechanical stretching and inflammation in vitro. (a)** For mechanical stress, neurons were inoculated in 6-well/24-well plates (BioFLEX®), and equiaxial stretch (0%, 6%, 8%, 12% or 16% strain, 1.0 Hz frequency) was applied to cultured neurons for 4 h, by a Flexcell® FX-5000™ Tension System (Flexcell, USA). Then cell damage was detected by CCK8 assay and LDH release. **(b)** For inflammation-induced model, neurons were incubation for 4 h with 10 ng/ml LPS and/or 5 mM ATP for 30 min. Then cell damage was detected by CCK8 assay and LDH release. Data are presented as the mean ± SEM. n=3. **p* < 0.05, *vs.* control group.

**
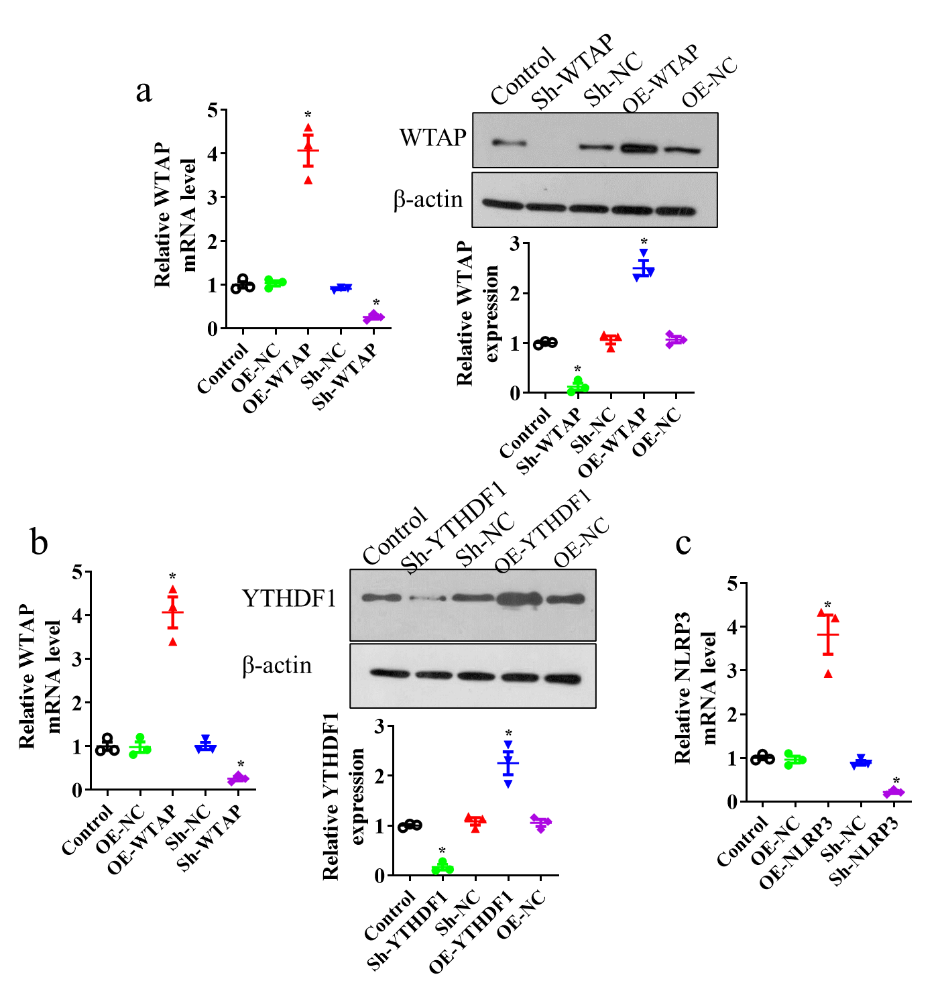
**

**Figure S4.** qRT-PCR and WB analysis of mRNA expression level in primary neurons after transfection. Primary microglia was infected with WTAP-shRNA/OE Lentiviral Particles, YTHDF1-shRNA/OE Lentiviral Particle, NLRP3-OE Lentiviral Particles, and the Control shRNA/Activation Lentiviral Particles (sc-108080, sc-437282) according to the manufacturer’s protocol (Santa Cruz Biotechnology, Inc.). WTAP (A), YTHDF1 (B), and NLRP3 (C) levels were measured. Data were represented as means ± SEM, n=3. **p* < 0.05, vs the control group.

**Figure S5 The full uncropped Gels and Blots images.**


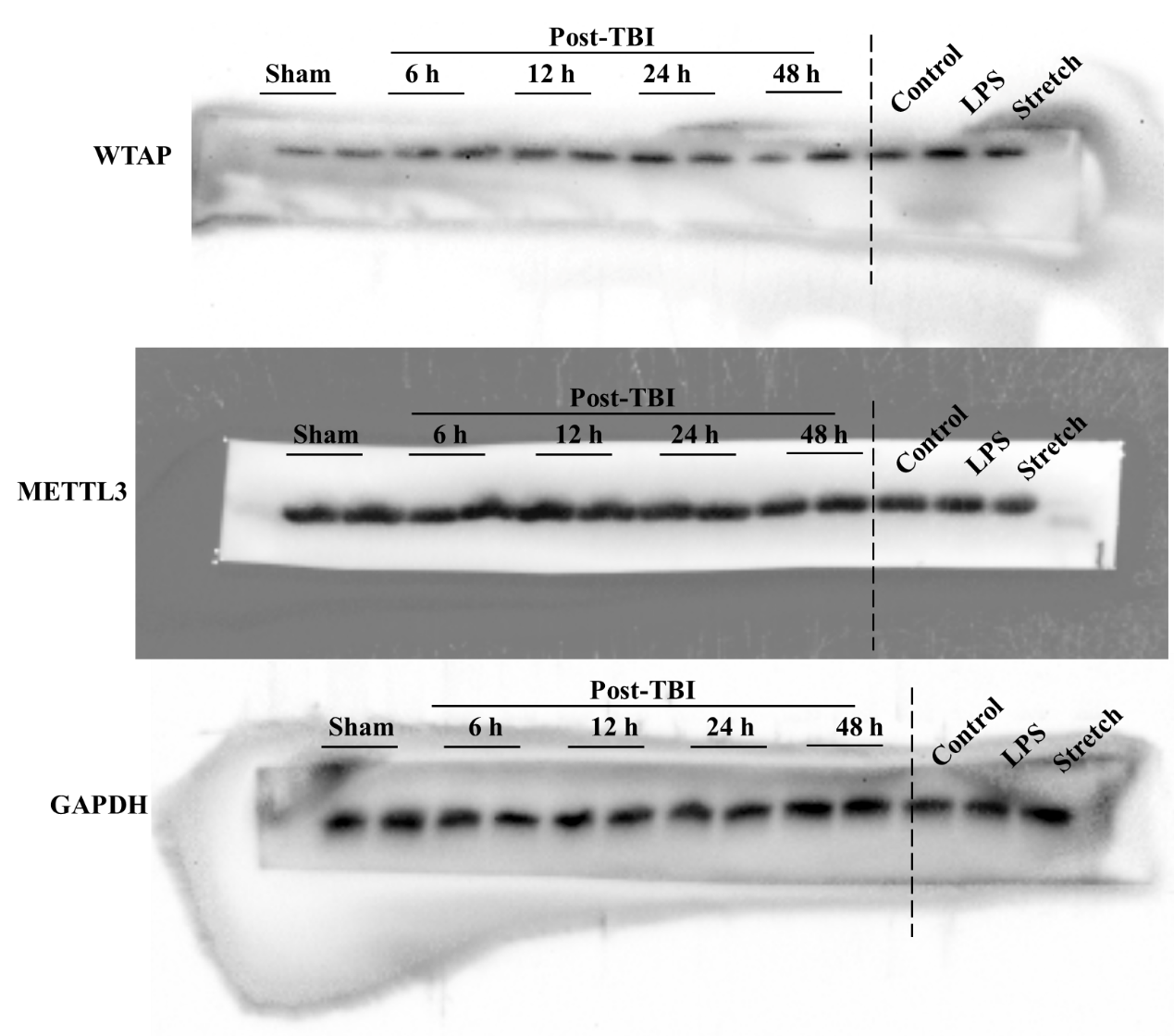


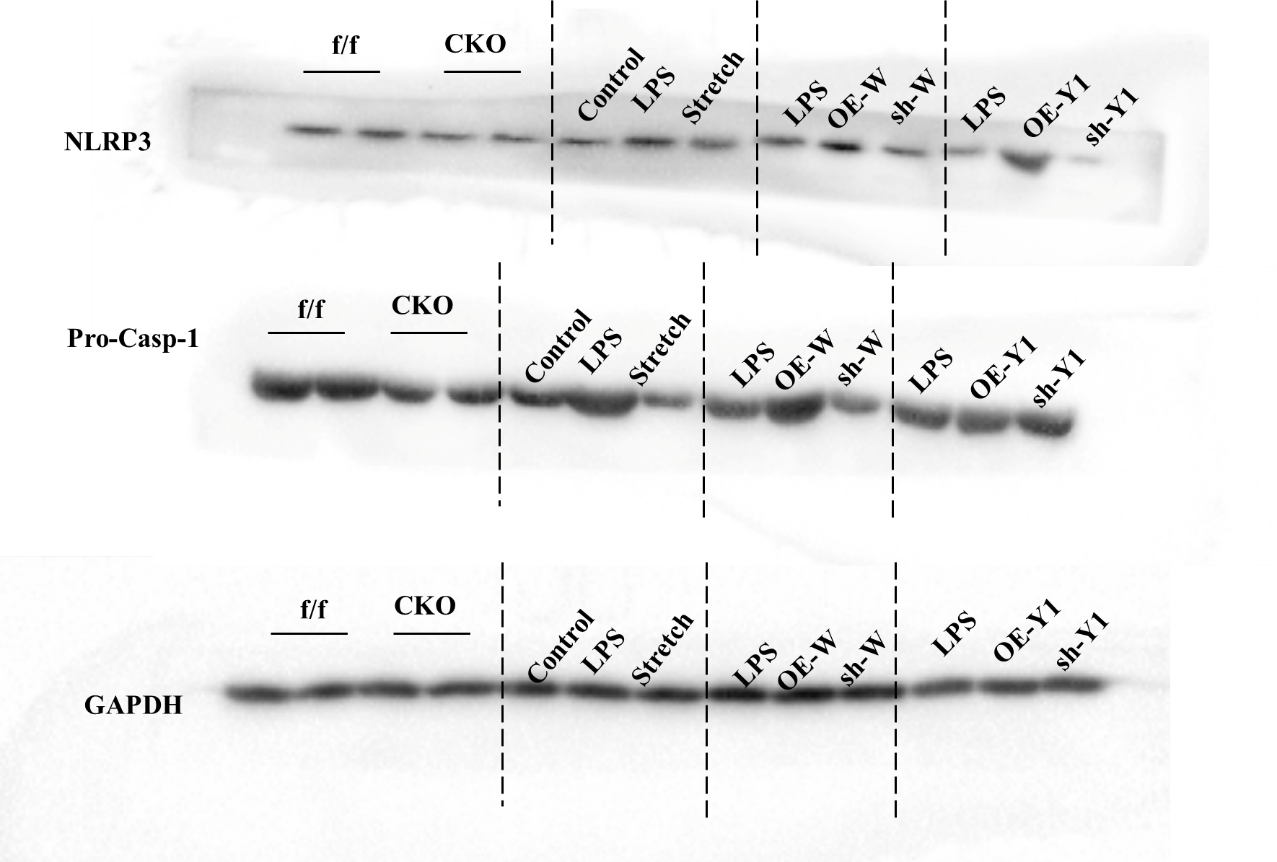


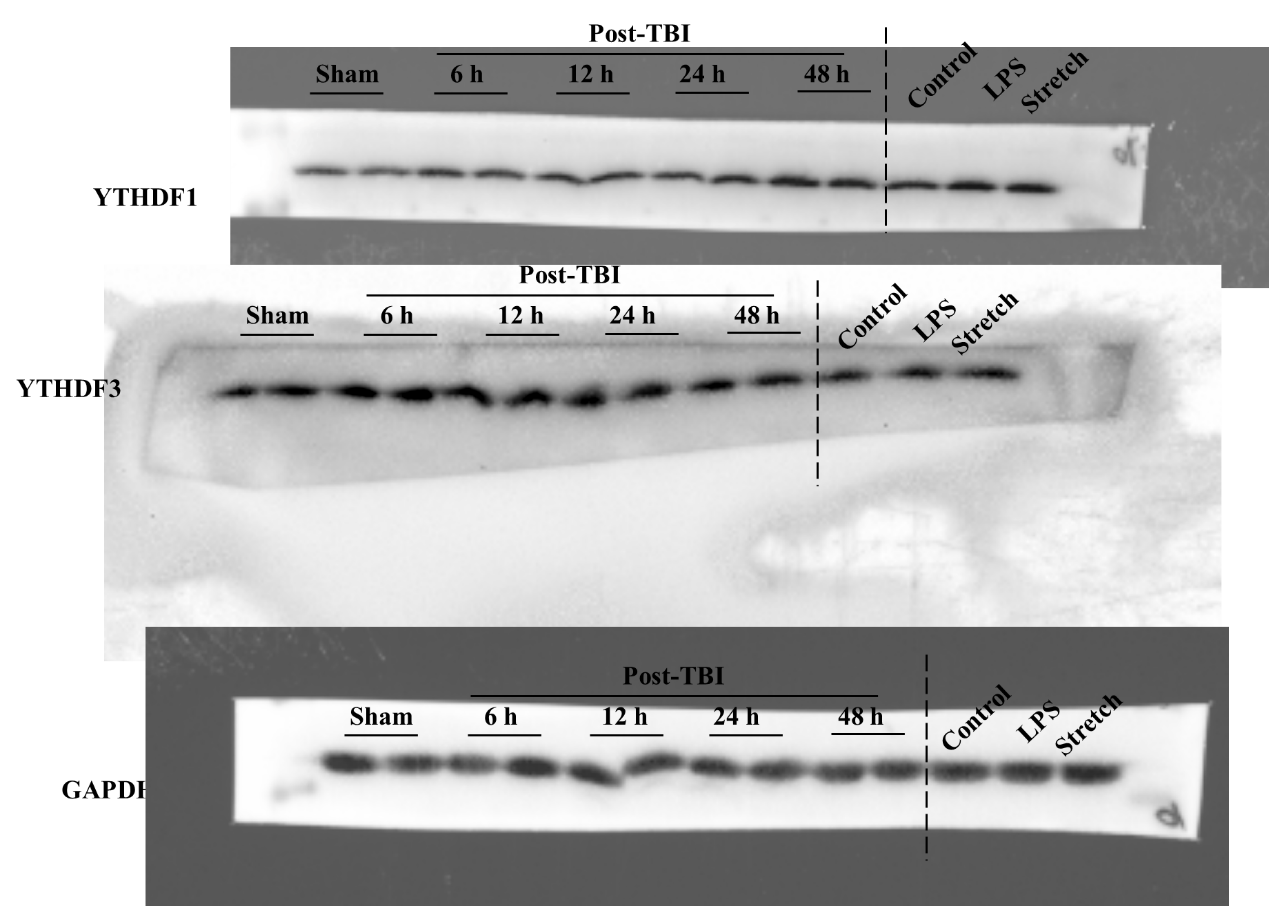


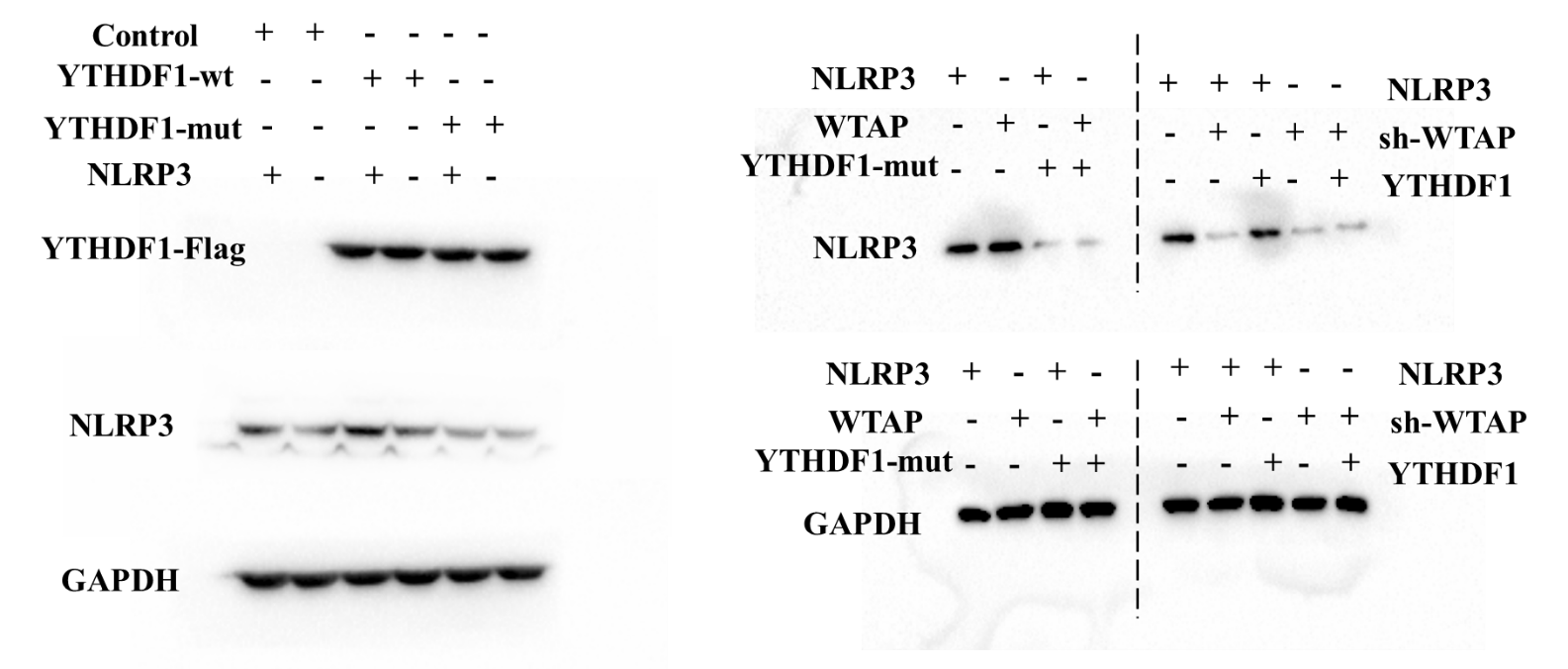

Supplement: Supplementary file 2 [file js9-110-5396-s002.docx]
